# Unconventional Edible Plants of the Amazon: Bioactive Compounds, Health Benefits, Challenges, and Future Trends

**DOI:** 10.3390/foods13182925

**Published:** 2024-09-15

**Authors:** Cynthia Tereza Corrêa da Silva Miranda, Stephanie Dias Soares, Williara Queiroz de Oliveira, Adriana de Souza Lima, Iramaia Angélica Neri Numa, Gláucia Maria Pastore

**Affiliations:** 1 Laboratory of Bioflavours and Bioactive Compounds, Department of Food Science, Faculty of Food Engineering, University of Campinas, Campinas 13083-862, SP, Brazil; s203084@dac.unicamp.br (S.D.S.); w229276@dac.unicamp.br (W.Q.d.O.); a216452@dac.unicamp.br (A.d.S.L.); iramaia@unicamp.br (I.A.N.N.); 2 Faculty of Pharmaceutical Sciences, Federal University of Amazonas—UFAM, Manaus 69080-900, AM, Brazil; 3 Faculty of Tourism and Hospitality, Federal Fluminense University—UFF, Gragoatá Campus, Niterói 24210-200, RJ, Brazil

**Keywords:** Amazon plants, biological activities, edible plants, green leaves, phenolic compounds, phytochemicals

## Abstract

The pursuit of an improved quality of life is a major trend in the food market. This is driving the reformulation of the industry’s product portfolio, with the aim of providing nourishment while also contributing to beneficial health metabolic processes. In this context, the use of local biodiversity and the recovery of the traditional knowledge associated with the consumption of vegetables that grow spontaneously in nature emerge as more sustainable and nutritionally adequate concepts. The Amazon region is known for its abundant biodiversity, housing numerous unconventional food plants whose nutritional and biological properties remain unknown due to a lack of research. Among the different species are *Xanthosoma sagittifolium*, *Acmella oleracea*, *Talinum triangulare*, *Pereskia bleo*, *Bidens bipinnata*, and *Costus spiralis*. These species contain bioactive compounds such as apigenin, syringic acid, spilanthol, and lutein, which provide various health benefits. There are few reports on the biological effects, nutritional composition, bioactive compounds, and market prospects for these species. Therefore, this review provides an overview of their nutritional contribution, bioactive compounds, health benefits, and current market, as well as the use of new technologies that can contribute to the development of functional products/ingredients derived from them.

## 1. Introduction

The search for a healthy diet has become an important goal for many consumers. A balanced diet includes an adequate amount of calories, essential nutrients, and micronutrients from different food groups. It is based on a wide variety of natural or minimally processed foods. It includes a minimum daily intake of five servings of fruits and vegetables. A healthy diet protects against malnutrition in all its forms, as well as against non-communicable chronic diseases such as diabetes, heart disease, strokes, and cancer [1]. Plant-based foods, such as fruits and vegetables, rich in essential nutrients and bioactive compounds, play a vital role in promoting optimal human nutrition and supporting overall well-being [2,3].

In this context, unconventional food plants (UFPs) are an excellent option to diversify the diet. In Brazil, they are known as PANCs (Plantas alimentícias Não-Convencionais), an acronym created by Kinupp [4]. UFPs can be defined as edible plant species with parts such as leaves, roots, stems, and flowers that have food potential but are not used daily [5]. They are part of the gastronomy of specific localities and can be consumed raw in salads and juices or in stews, jelly, and sweets. Some of these species are potential sources of nutrients and bioactive compounds, while others are used for medicinal purposes. In addition to their potential contribution as nutraceuticals, these plants are easy to cultivate and adapt to the environment as they do not require pesticides or fertilizers [6].

UFPs have significant economic potential and can support healthier and more sustainable diets, as well as boost family farming [6]. Studies by the Brazilian Institute of Geography and Statistics (IBGE) have shown that the primary consumers of UFPs are women with an average income of USD 293.02, suggesting a great potential for including these plants in the Brazilian diet. On the other hand, in rural areas, the main consumers are people with lower incomes who rely on natural resources for food, where UFPs are essential to avoid food insecurity [7,8].

The Amazon region has one of the planet’s most outstanding plant genetic diversities, with around 15,000 species [9]. Many UFPs are part of this biome (Table 1); however, few are included in the population’s diet. A study revealed that only “taioba” (*Xanthosoma taioba*) and “tucumã” (*Astrocaryum aculeatum*) are among the Amazonian UFPs consumed by the Brazilian population [7]. The lack of knowledge about the potential of these species is a factor contributing to their underutilization. Conducting phytochemical studies and in vitro and in vivo assays can reveal their potential, stimulate the consumption of these species, and boost the local economy.

One example is açaí (*Euterpe oleracea*), considered a “superfruit” due to its high antioxidant capacity from the presence of anthocyanins, proanthocyanidins, and other flavonoids [10]. Additionally, it has already demonstrated biological properties such as hepatoprotective [11], anti-inflammatory [12], neuroprotective [13], and wound healing [14]. The recognition of the benefits that this fruit can promote to health has increased its production and exportation, so that today it is possible to find products made with açaí sold internationally in the form of purees, juices, and dietary supplements [10].

**Table 1:** Some examples of UFPs consumed in the Amazon, their respective parts used, and the forms of consumption.

Botanical Family	Scientific Name	Common Name *	Parts Used	Forms of Consumption
Arecaceae	*Euterpe oleracea* Mart.	Açaí	Fruit	Raw, juice, wine, desserts, ice cream, and sauces
Malvaceae	*Hibiscus sabdariffa*	Vinagreira	Calyx and leaves	Soups, sauces, beverages, jams, and jellies
Malvaceae	*Pachira aquatica*	Monguba	Fruit pericarp, seeds, fruit, trunk, leaves, and flowers	Raw and used in confectionery, bakery, oils, and biofuels
Clusiaceae	*Garcinia gardneriana*	Bacopari, bacupari, abricó, and damasco	Fruit	Raw, juice, jams, and jellies
Fabaceae	*Inga marginata* Willd.	Angá-feijão, ingá-feijão, and angá	Fruit and seeds	Raw
Myrtaceae	*Psidium cattleianum* Sabine	Araçá, araçá-amarelo, araçá-roxo, araçá-vermelho, and araçá-manteiga	Fruit	Raw, juice, jams, and jellies
Marantaceae	*Goeppertia allouia*	Ariá	Tuber	Cooked or used as ingredients in culinary preparations
Arecaceae	*Bactris gasipaes*	Pupunha	Fruit pulp, peel, and seeds or stem	Cooked pulp, flour from pulp and peel, as a source of starch, in oil production, and heart of palm
Apiaceae	*Eryngium foetidum*	Chicória-do-Pará, chicória paraense, and coentrão	Leaves	Used as a condiment or seasoning for fish and as an ingredient in the preparation of typical meals, stews, omelets, and stir-fries
Humiriaceae	*Endopleura uchi* (Huber) Cuatrec	Uxi and uxipuçu	Pulp, peel, seed, and bark	Raw, tea, ice cream, and sweets; and in skincare products and as a powerful insecticide

Notes. * In Brazil. References: [15,16,17,18,19,20,21,22,23,24,25].

Many Amazonian species are underexplored and lack bioprospecting studies that reveal their nutritional importance, biological properties, and health benefits for use in the food, chemical, and pharmaceutical industries [5,6]. This is the case for species such as *Pereskia bleo*, *Bidens bipinnata*, and *Costus spiralis*. Other plants such as “jambu” (*A. oleracea*) and “taioba” (*X. sagittifolium*) also require further investigation, and few studies have already demonstrated their applications as functional food ingredients and the components of edible films for food [26,27,28].

**Figure 1:**
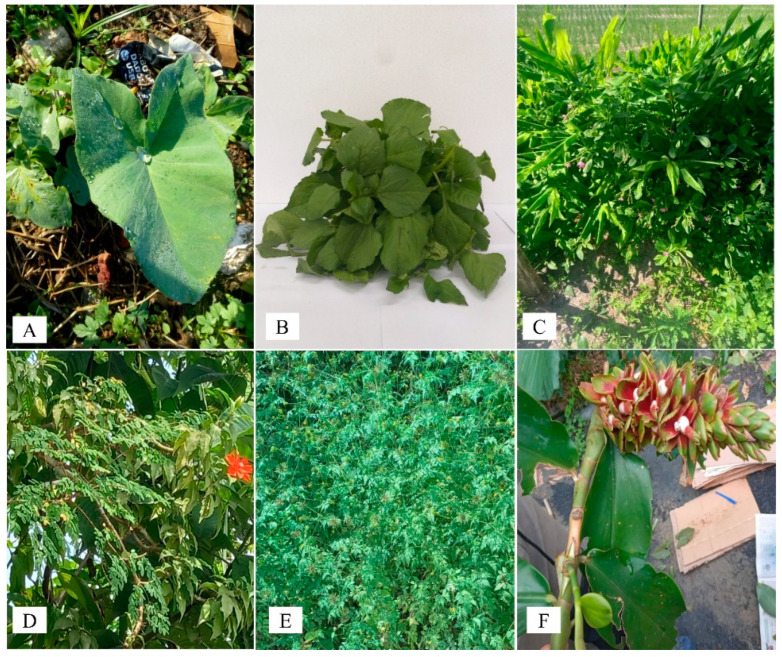
Unconventional food plants commonly found at fairs and markets in the Amazon region: (**A**) *Xanthosoma sagittifolium*; (**B**) *Acmella oleracea*; (**C**) *Talinum triangulare*; (**D**) *Pereskia bleo*; (**E**) *Bidens bipinnata*; and (**F**) *Costus spiralis*. Source: Natália Santos Reis da Cunha (**A**), Sebastião Rebelo de Miranda (**B**), and Cynthia Tereza Corrêa da Silva Miranda (**C**–**F**).

**Figure 2:**
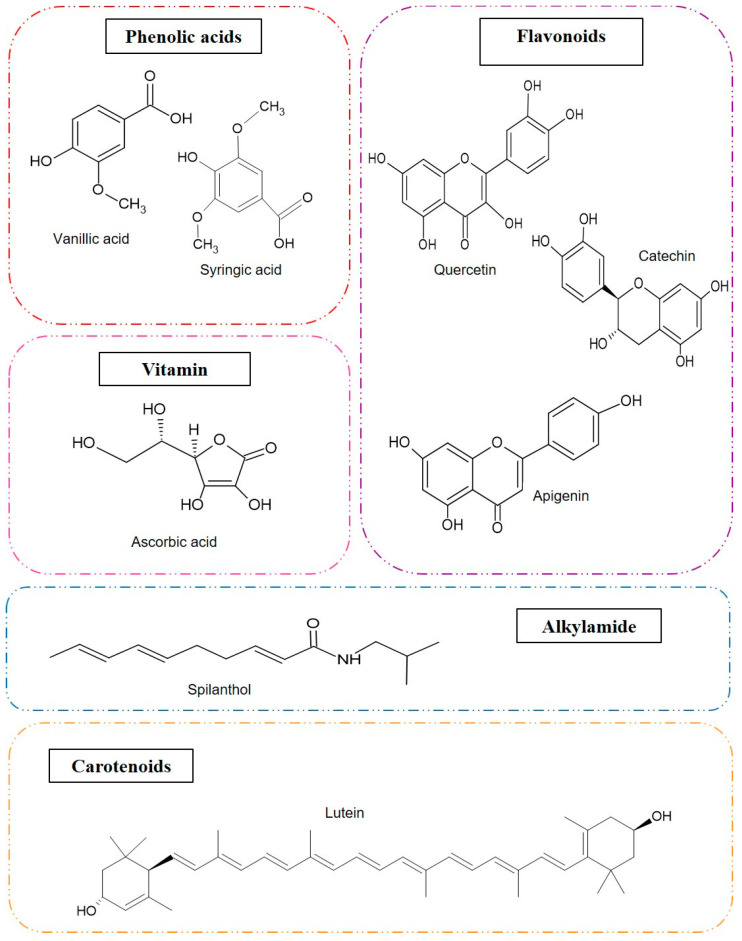
Chemical structure of some bioactive compounds found in unconventional vegetables from the Amazon region.

Therefore, this review aims to discuss the main research conducted on the Amazonian UFPs *Xanthosoma sagittifolium*, *Acmella oleracea*, *Talinum triangulare*, *Pereskia bleo*, *Bidens bipinnata*, and *Costus spiralis* (Figure 1). The major bioactive compounds (some of which are shown in Figure 2) and biological properties and the use of technologies that can add value to these species, as well as the challenges and trends associated with the consumption of UFPs, will be addressed, revealing existing knowledge gaps and insights for future research.

## 2. Chemical Composition and Biological Effects of UFPs Consumed in the Amazon

### 2.1. Xanthosoma sagittifolium (L.) Schott

*Xanthosoma sagittifollium*, also known as “taioba” and “arrow leaf elephant ear” or just “elephant ear leaf”, is a species belonging to the Araceae family, originally from Central America. Nevertheless, today it is widespread and consumed in several countries, including Brazil [29]. These leaves (Figure 1A) weigh approximately 48 g and are about 35 and 48 cm in transverse and longitudinal lengths, respectively [30]. Nevertheless, these physical characteristics can be easily modified according to the cultivation system [31] and planting site [32].

Due to their morphological similarities, *X. sagittifollium* leaves can be easily confused with those of the yam (*Colocasia esculenta*), so it is essential to distinguish them correctly. The *X. sagittifollium* species has a petiole directly connected to the “lobes” of the plant; in addition, the green color of the leaves and petiole is uniform in tone, and the veins of the leaves have a slight yellow color [30,33]. *X. sagittifollium* (including its rhizome and corms) is acrid and leads to the swelling of the lips, mouth, and throat due to the raphides of calcium oxalate, which it causes. Therefore, it needs to be cooked before consumption to remove the irritating compounds [33,34].

*X. sagittifollium* leaves are used by the local population in culinary preparations such as stews and soups, being braised with spices and seasonings [35,36]. According to Barbosa et al. [36], this plant is used as a value-added food to supplement meals at a school in the northeast region of Brazil. Concerning its use in traditional medicine, the tuber part is an antimalarial agent [37], and its tea has antidiarrheal properties [38]. In addition, the leaves’ exudate can potentially manage skin cancer [39].

A summary of the nutritional composition of *X. sagittifollium* can be seen in Table 2. *X. sagittifollium* leaves can be considered excellent sources of minerals, such as calcium > magnesium > phosphorus > iron > zinc [34]. This study corroborates the study of Silva et al. [40], who assessed the nutritional composition of ten non-conventional vegetables in Brazil and detected the highest potassium, copper, and phosphorus levels for *X. sagittifollium*. On the other hand, Moura et al. [41] found higher levels of phosphorus (323.9 mg/100 g) compared to calcium (135.1 mg/100 g) and magnesium (109.6 mg/100 g) for *X. sagittifollium* leaves. These studies demonstrate that this vegetable has a mineral composition highly influenced by location, climate, soil, and crop. These highlighted minerals are essential to bone tissue, cardiovascular, and skeletal muscle health [42,43].

The content of some bioactive compounds in the *X. sagittifollium* leaf was assessed, such as the ascorbic acid and phenolic compounds, both of which are directly related to the antioxidant potential of vegetables [44,45,46]. This vegetable has a high ascorbic acid content (195.58 mg/100 g) [40] and the active form of vitamin C (dehydroascorbic acid) with a content of 63.99 mg of ascorbic acid/100 g [47]. Ascorbic acid is directly related to preventing scurvy by incorporating O_2_ into the substrate of enzymatic reactions for collagen synthesis, reducing Fe^3+^ to Fe^2+^, which keeps the enzymes active [48]. Ascorbic acid also acts as an essential agent in combating tumor cells [49], reducing mortality due to septic shock [50], and bringing about cognitive improvement [51].

Moncayo and Boudjeko [52] found abundant concentrations (values not shown) of alkaloids, flavonoids, steroids, and terpenoids in the ethanolic extract of the *X. sagittifollium* leaf through a phytochemical screening. Likewise, this plant can also be a good source of total phenolics (24.15 mg GAE/g) and flavonoids (17.15 mg/100 g) [30]. The aqueous extract of this leaf had the flavone metabolites apigenin (Figure 2), isovitexin, and vitexin [53]; however, Moura et al. [41] found 522.00 µg/g of syringic acid (Figure 2) and 379.00 µg/g of caffeic acid as the most prominent phenolic compounds in the acidified methanol extract.

In general, there are few studies on the biological properties of *X. sagittifollium* leaves. This scarcity can be explained by the underuse of its leaves [54] compared to its corm, specifically in the functional properties of starch [55]. Previously, the hydroethanolic extract of *X. sagittifolium* leaves, rich in di-C-glycosides, had an antileukemic effect and was capable of inhibiting cell proliferation by 50.3% [56]. Furthermore, the ingestion of the lyophilized leaf by Wistar rats was able to improve intestinal health due to its high content of insoluble fiber, which provided the insight to consider that this vegetable could contribute to a reduction in the risk of colon cancer [57]. Therefore, *X. sagittifollium* leaves demonstrate a potential antiproliferative effect. Other biological activities can be seen in Table 3.

### 2.2. Acmella oleracea (L.) R. K. Jansen

*Acmella oleracea*, also popularly known as “jambu”, “agrião-do-Pará”, “agrião-do-norte”, “agrião-do-Brasil”, and “jambuassu”, is an autochthonous plant commonly found in the Amazon region, mainly in Brazil, Colombia, Guianas and Venezuela, where it can be found cultivated or subspontaneously. There are also reports of its cultivation in tropical regions near the equator in Africa, Asia, and South America [58,59]. It is one of the most distinguished members of the *Acmella* genus, being a small herbaceous plant with creeping, branched stems. It has long petiolate leaves, oppositely arranged, ovate, toothed, and with an acute apex (Figure 1B). Yellow flowers are arranged in capitula, terminal, or axillary. The fruit is an achene, oblong, margined, and aristate, and the seeds are flattened and small [58,59].

In the Amazon region, *A. oleracea* is widely used in cuisine and folk medicine, being beloved due to its slightly spicy flavor and served in typical dishes such as “tacacá” and “pato no tucupi”, in addition to being part of salads, rice, and beers. *A. oleracea* brings a curious sensation of numbness in the mouth caused by the spilanthol, an alkaloid that, in addition to tingling, stimulates salivation and increases appetite [59]. At the same time, local knowledge and practices make use of the fresh plant, whether in the form of teas, syrups, and tinctures or prepared from the leaves or flowers for treating toothaches, anemia, scurvy, dyspepsia, bladder stones, and liver and respiratory problems. In addition, hydroethanolic formulations are popularly used as a female aphrodisiac and for male sexual dysfunctions [58,60].

The nutrients and non-nutrients present in *A. oleracea* are shown in Table 2. Anju et al. [61] highlighted the nutritional importance of *A. oleracea* leaves considering the crude fiber content (8.42%) and proteins (10%), which are similar to those of the other 27 leafy vegetables investigated by Arumugam et al. [62], being higher in proteins than Amaranthus (6.57%). Regarding the phytochemistry profile, several polyphenols (e.g., vanillic, trans-ferulic, trans-isoferulic acids, and scopolentin) and fatty acids (n-hexadecanoic and n-tetradecanoic acids) have been found [60]. In this sense, its biological potential is characterized by the presence of secondary metabolites such as N-alkylamides (mainly spilanthol, shown in Figure 2), triterpenoids, and phytosterols [60,63].

Most of the reported studies correlate the in vitro (e.g., DPPH, ABTS, FRAP, ORAC, etc.) antioxidant potential with the presence of the aforementioned secondary metabolites. For example, Nascimento et al. [64] evaluated the antioxidant capacity of the leaves, flowers, and stems of *A. oleracea* cultivated in hydroponic and conventional systems by ABTS and FRAP assays. As a result, the leaves have the highest antioxidant capacity (9.43 mM TE/g (trolox equivalent) and 10.77 mM TE/g, respectively), probably due to components such as phenolics and flavonoids. Another similar study also reported that the leaves exhibit the highest levels of DPPH and ABTS (8.60 µmol TE/g and 4.53 µmol TE/g, respectively) [65].

Regarding the biological effects, *A. oleracea* has been associated with numerous functions, including analgesic, anti-inflammatory, antioxidant, antimicrobial, and toothache relief [59,63,66], among the others shown in Table 3. A previous study reported the beneficial effects of its crude extract on tendon repair through the molecular organization and content of collagen [67], while another observed that isolated spilanthol (the most studied bioactive of *A. oleracea*) might exert anti-obesity effects by the upregulation of mitogen-activated protein kinase attenuating both lipogenic and adipogenic transcription factors [68]. Recently, Radhika et al. [69] evaluated whether the *A. oleracea* extracts’ nanostructured Ca_2_Fe_2_O_5_ may be used as a drug for wastewater treatment purposes. As a result, the authors highlighted the application possibilities in bioremediation, particularly in degrading cardiovascular pharmaceutical pollutants, endodontic antibacterial action, and cytological activity.

**Table 2:** Nutrient and non-nutritive composition of taioba (*Xanthosoma sagittifolium*), jambu (*Acmella oleracea*), cariru (*Talinum triangulare*), ora-pro-nóbis (*Pereskia bleo*), and pobre-velho (*Costus spiralis*) leaves.

Composition	*Xanthosoma sagittifolium* ^1^	*Acmella oleracea* ^2^	*Talinum triangulare* ^3^	*Pereskia bleo* ^4^	*Costus spiralis* ^5^
Moisture	88.58 g/100 g and 93.86 g/100 g (petiole) ^b^	89.87 g/100 g ^a^	87.13 g/100 g ^a^	91.39 g/100 g ^b^	-
Ash	13.77 g/100 g and 22.12 g/100 g (petiole) ^a^	1.11 g/100 g ^a^	7.92 g/100 g ^a^	2.15 g/100 g ^b^	-
Lipids	7.60 g/100 g and 5.86 g/100 g (petiole) ^a^	0.16 mg/100 g ^a^	1.98 g/100 g ^a^	0.41 g/100 g ^b^	-
Proteins	58.50 g/100 g and 30.90 g/100 g (petiole) ^a^	2.44 mg/100 g ^a^	14.65 g/100 g ^a^	3.25 g/100 g ^b^	-
Soluble fibers	3.50 g/100 g ^a^	-	-	-	-
Insoluble fibers	11.55 g/100 g ^a^	-	-	-	-
Total fibers	23.39 g/100 g and 16.66 g/100 g (petiole) ^a^	6.35 mg/100 g ^a^	7.92 g/100 g ^a^	-	-
Sodium	129 mg/100 g ^a^	1.62 mg/100 g ^a^	31.00 mg/100 g ^a^		-
Potassium	3.03 g/100 g ^a^	594.44 mg/100 g ^a^	3546.00 mg/100 g ^a^	619.50 mg/100 g ^a^	1.40 mg/Kg ^a^
Calcium	1.79 g/100 g and 0.98 g/100 g (petiole) ^a^	260.00 mg/100 g ^a^	678.00 mg/100 g ^a^	480.71 mg/100 g ^a^	27.30 g/Kg ^a^
Magnesium	0.50 g/100 g and 0.25 g/100 g (petiole) ^a^	74.86 mg/100 g ^a^	1983.00 mg/100 g ^a^	88.27 mg/100 g ^a^	11.80 g/Kg ^a^
Phosphorus	41.99–43.89 mg/100 g ^a^	-	436.00 mg/100 g ^a^	-	3..50 g/Kg ^a^
Iron	7.22–7.89 mg/100 g ^a^	1.94 mg/100 g ^a^	14.33 mg/100 g ^a^	12.34 mg/100 g ^a^	545.00 mg/Kg ^a^
Zinc	4.15–4.60 mg/100 g ^a^	0.95 mg/100 g ^a^	4.24 mg/100 g ^a^	6.40 mg/100 g ^a^	22.00 mg/Kg ^a^
Calcium oxalate	648 mg/100 g and 846.72 mg/100 g (petiole) ^a^	-	-	-	-
Vitamin C	87 mg/100 g and 83.00 mg/100 g (petiole) ^a^	-	1.11–1.36 g/100 g ^a^	-	-
Total phenolics	5.33 mg GAE/100 g and 2.80 mg GAE/100 g (petiole) ^a^	3.19 g GAE/g ^a^	0.61–1.09 g GAE/100 g ^a^	109.43 mg GAE/g	-
Total flavonoid	-	11.45 mg rutin/g ^a^	0.33–3.52 g/100 g ^a^	-	-
Total CHL	8.94 mg/100 g and 7.00 mg/100 g (petiole) ^a^	-	92.26–584.19 g/100 g ^a^	-	-
Tannins	1.08–1.11 mg/100 g ^a^	-	-	-	-
Total carotenoids	83.19 mg/100 g and 54.07 mg/100 g (petiole) ^a^	618.00 μg/g ^a^	-	-	-

Notes. GAE: gallic acid equivalent; and CHL: chlorophyll content. ^1^ [30,34,70,71]; ^2^ [64,72]; ^3^ [73,74,75]; ^4^ [76,77]; and ^5^ [78]. ^a^ Values expressed on the basis of dry weight; and ^b^ values expressed on the basis of fresh weight.

### 2.3. Talinum triangulare

The tropical herbaceous dicot plant *Talinum triangulare* (Figure 1C) is recognized as a synonymous species of *Talinum fruticosum*, which belongs to the Talinaceae family (previously known as Portulacaceae) [79]. *T. triangulare*, known as “cariru”, “joão-gomes”, and “major-gomes”, is traditionally from the Amazon region and is used in popular Brazilian medicine or food [18]. The availability of vegetables, such as *T. triangulare,* has declined due to the changes in food habitats and the rise in other crops [73]. This plant is a wild leafy vegetable with a protein content equivalent to legume seeds, as well as being low-fat and high-fiber and containing carbohydrates.

Considering the minerals listed in Table 2, this leaf can be identified as a rich source of potassium and magnesium, meeting the dietary needs for both children and adults with favorable sodium/potassium and calcium/phosphorus ratios. Cooked *T. triangulare* showed improved protein digestibility, protein-corrected amino acid score, protein efficiency ratio, and total unsaturated fatty acids, making it suitable for addressing protein energy malnutrition. Several processing options, such as blanching, boiling, frying, and microwaving, can be used to prepare fortified foods that can combat lifestyle-related diseases without compromising its nutraceutical potential [80]. In the *T. triangulare* leaves, the vitamin concentrations (in mg/100 g) were 30.16 for ascorbic acid, 0.96 for riboflavin, 0.11 for thiamine, and 2.89 for niacin, along with 112.25 μg/100 g of vitamin K [81]. In general, the composition of bioactive compounds in *T. triangulare*, such as phenolics and carotenoids, is relatively unexplored, particularly concerning their levels.

The carotenoid compounds of *T. triangulare* consisted of violaxanthin, lutein (Figure 2), zeaxanthin, isomers of β-carotene (trans-β-carotene and cis-β-carotenes), and others [74]. According to Okpalanma and Ojimelukwe [81], lutein (124.03 µg/g) was the major carotenoid in raw leaves and increased after cooking (593.24 µg/g). This was followed by 45.42 µg/g of β-carotene isomers and 5.11 µg/g of β-cryptoxanthin.

Brasileiro et al. [73] found in the *T. triangulare* leaves and stems the presence of phytochemicals such as alkaloids, flavonoids, coumarins, and triterpenes, which were specifically identified in the leaf, and steroids, which were identified in the stem. These results were subsequently reported and quantified by Amusat et al. [75]. The authors found saponins at 0.99 mg/100 g, alkaloids at 7.93 mg/100 g, and phytates at 9.76 mg/100 g. The phenolic and flavonoid compounds identified and quantified (contents not shown) using liquid chromatography were catechin (Figure 2), protocatechuic acid, gallic acid, rutin, quercetin (Figure 2), ferulic acid, para-coumaric acid, and trans-cinnamic acid [74].

The biological activities of *T. triangulare* have been the subject of investigations, like those shown in Table 3 and many others. A notable increase in the blood parameters (including the hematocrit, hemoglobin, red blood cell count, mean corpuscular volume, leukocyte count, lymphocyte count, neutrophil count, and platelet count) was noted in animals treated with 100 mg/kg of leaf extract for 28 days. Thus, the leaves of *T. triangulare* possess hematopoietic properties and can be utilized to enhance blood levels, particularly in menstruating and pregnant women and individuals with anemia. Moreover, the notable increase in the white blood cell parameters suggests that *T. triangulare* leaves may enhance the immune system, providing protection against harmful substances [82].

*T. triangulare* leaves are highly beneficial in managing conditions such as hyperglycemia and hyperlipidemia, which are associated with high blood sugar and lipid levels. During a 14-day trial, Wistar rats that were given *T. triangulare* leaf extract showed a significant decrease in fasting blood sugar, total cholesterol, LDL cholesterol, and tri-glycerides. Additionally, there was a noteworthy increase in HDL cholesterol levels and the HDL/LDL–cholesterol ratio compared to the control group [83]. Oluba et al. [84] corroborated these results by administering *T. triangulare* leaf flavonoid extract for 21 days. The authors discovered that the extract normalized streptozotocin-induced hyperglycemia and its associated dyslipidemia through several mechanisms. These mechanisms included enhanced plasma insulin secretion, which could stimulate cellular glucose uptake and the inhibition of α-amylase activity, thereby regulating the release of glucose into the blood, as well as the regulation of hepatic lipid synthesis via the inhibition of 3-hydroxy-3-methylglutaryl-CoA reductase activity in rats.

Mathala et al. [85] found that the hydroalcoholic extract of *T. triangulare* leaves showed a dose-dependent neuroprotective effect through both in vitro and in vivo antioxidant activities. This was evidenced by the increased levels of superoxide dismutase and catalase in the ischemia/reperfusion brain. In a study model of ethanol-induced oxidative stress, these leaves were able to mitigate this effect by regulating oxidative stress biomarkers [86]. More recently, Afolabi et al. [87] assessed the antioxidant activity and the neuroprotective potential of the leaf’s aqueous extract. The authors found that there was a significant concentration-dependent inhibition against ABTS cation radicals. Additionally, simulation and molecular docking analyses revealed that rutin and quercetin have strong binding energies for acetylcholinesterase and butyrylcholinesterase. These enzymes are particularly important for maintaining acetylcholine and its activity at the cholinergic synapses for normal cognitive function in dementia-related diseases.

The extracts from these UFPs show significant potential in reducing oxidative stress and improving cognitive function, emphasizing their importance in developing therapeutic strategies for related health conditions.

### 2.4. Pereskia bleo

*Pereskia bleo*, also known as “*ora-pro-nóbis*”, is a plant belonging to the Cactaceae family and can grow up to 8 m in height. The leaves are radiant green and broad and have long, spiny stems. It has thorns in fascicles of five to six, and its flowers or buds can be seen singly or in clusters. The flowers change from white and yellow to fuchsia or red (Figure 1D). The fruits are generally round and green and change to yellow when ripe [88]. It originates from South America and can be found in countries like Malaysia, Indonesia, Singapore, and India [89]. *P. bleo*, like other species of this genus, has also been used as food, being an important source of nutrients, as described in Table 2.

Mohd-Salleh et al. [90] investigated the phytochemical profile in several leaf extracts and identified terpenoids, sterols, phenols, alkaloids, and fatty acids, with an emphasis on the latter, which were higher in the methanolic (9.8%) and aqueous (5.51%) extracts of *P. bleo* leaves. The total phenolic compounds content was determined in the methanolic extract, corresponding to 40.82 mg GAE/g [89].

Some in vitro and in vivo studies were performed to evaluate the biological properties of *P. bleo*. They point out that the species has hypoglycemic, antibacterial, antihypertensive, and antiproliferative activities. The aqueous extract of *P. bleo* leaves significantly reduced blood glucose and the levels of cholesterol, triglycerides, and LDL-c in male streptozotocin-induced diabetic Sprague Dawley rats, and it was observed that there was a significant restoration of serum insulin in diabetic rats, which would probably also regulate the flow of fatty acids [91].

The aqueous extract of the leaves has also been evaluated for anticancer activity. The extract exhibited a high cytotoxic activity with an IC_50_ value of 14.37 µg/mL selectively against HeLa cells (cervical cancer cells), demonstrating high anticancer potential through the Bax/Bcl-2 signaling pathway with the involvement of caspase-3, which are important elements in apoptosis [90]. These results were corroborated by Siew et al. [92], who also found antiproliferative activity of the leaf extract (2 mg/mL) in the cell lines from the breast (T47D), cervical (C33A), colon (HCT116), liver (SNU-182, SNU-449, HepG2), ovary (PA-1), and uterine cancer cells (MES-SA/Dx5).

Siska et al. [93] investigated the antihypertensive effect of the oral administration of *P. bleo* extract (PBE) in sodium chloride-induced hypertensive male rats and they observed a reduction in blood pressure and an increase in the urinary sodium and potassium levels. According to the authors, the polyphenolic compounds of PBE can increase the production of nitric oxide, leading to the activation of eNOS mRNA expression and promoting the relaxation of blood vessels.

Research points to *P. bleo* as a potentially nutritious food source with beneficial effects on health. However, more robust evidence is needed to identify its phytochemical constituents, including deeper investigations into its biological effects, as well as the mechanisms involved.

### 2.5. Bidens bipinnata L.

*Bidens bipinnata* L. (Figure 1E) is an herb belonging to the Asteraceae family; it is easy to cultivate and has been used in traditional Chinese folk medicine to treat various diseases, such as hyperlipidemia, hypertension, diabetes, malaria, inflammation, and liver fibrosis [94]. In Brazil, it is distributed in all regions and is popularly known as “picão-preto”, “beijo-de-moça”, and “carrapicho-de-agulha” [95].

The nutritional composition of *B. bipinnata* is not well studied, so Table 2 does not include these data. However, research has been conducted on its phytochemical composition. Studies on its phytochemical composition based on MS and NMR spectroscopic data revealed the presence of ceramides, flavonoids, phenylpropanoids, aliphatics, one pyrimidine, steroids, one triterpenoid, and one polyacetylene in the plant extracts and fractions [96]. Later, Yang et al. [97] developed a simple and efficient method for enriching total flavonoids from *B. Bipinnata*, using an AB-8 resin for the purification of the crude extract. Fourteen compounds were identified, including flavonoids such as rutin, isoquercitrin, quercetin 3-O-β-d-glucuronide, quercitrin, 4,5-dicaffeoylquinic acid, luteolin, isookanin 7-O-D-(2″,4″,6″-triacetyl)-glucopyranoside, and okanin 4′-O-D-(2″,4″,6″-triacetyl)-glucopyranoside.

Some studies have already investigated some biological properties of *B. bipinnata*. Tests on male Sprague Dawley rats treated with its extracts showed a significant reduction in total cholesterol, triglycerides, and LDL-c levels and a significant increase in HDL-c levels. The biochemical parameters related to the kidney and liver function (creatinine, bun, aspartate aminotransferase, alkaline phosphatase, and alanine aminotransferase) were also significantly reduced. Finally, the authors concluded that the extracts exerted a significant improvement in the lipid levels, liver function, kidney function, and the mRNA expression level of the PPARs signaling pathway, which acts in the regulation of lipid and glucose metabolism, energy homeostasis, blood pressure control, and cell proliferation and differentiation [98]. These results were corroborated by Li et al. [99] in hyperlipidemic rats.

*B. bipinnata* has been used as a decoction in treating diabetes mellitus in different regions of the world for a long time, and studies show that flavonoids are considered the main compounds associated with antidiabetic activity. The effects of the flavonoid-rich extract of *B. bipinnata* were evaluated on H_2_O_2_-induced apoptosis in INS-1 cells (rat pancreatic β cells), and the production of the ROS induced by H_2_O_2_ was attenuated, demonstrating the protective effect against cell apoptosis, which can be attributed to the antioxidant activity of the plant [100]. Other biological activities related to this species are listed in Table 3.

**Table 3:** A summary of the in vitro and in vivo studies of the biological potential of Brazilian unconventional food plants.

Unconventional Food Plant	Source	Bioactivity	Method/Model	Related Compounds	Major Findings	References
*Xanthosoma sagittifolium*	Methanolic extract of the whole plant	Antioxidant	DPPH, ABTS, and FRAP assays	Not informed	-High antioxidant capacity in the DPPH (4173 g/g), ABTS (33.55 µM TE/g), and FRAP (0.0144 µM FS/g) assays;	[71]
	Methanolic extract of the leaves	Antioxidant and antiproliferative	ORAC and HOCl assays	Not informed	-High antioxidant capacity in the ORAC (632.26 µM TE/g) and HOCl (35.21 µg/mL) assays. -Inhibited cell proliferation of human tumor cell lines (GI50): glioblastoma (205.1 µg/mL), melanoma (225.7 µg/mL), ovarian II (185.6 µg/mL), kidney (116.0 µg/mL), and leukemia (13.9 µg/mL).	[47]
	Methanolic extract of the corm	Anti-hypertensive	Forty normotensive male Wistar rats with induced hypertension from II of DOCA salt twice weekly and the daily inclusion of NaCl (1%) in their drinking water. The rats received 100 or 200 mg/kg of the extract	Not informed	-↓ in blood pressure and free protein thiols; -↓ in malondialdehyde levels and hydrogen peroxide activities; -↑ in total protein, gluthathione peroxidase, reduced glutathione, glutathione S-transferase, catalase, and nitric oxide in the heart, kidney, and liver.	[101]
*Acmella oleracea*	Leaves’ essential oil by hydrodistillation	Cytotoxicity	MTT test	*E*-Caryophyllene	-Antiproliferative activity against cell lines of human cancer: gastric ascites (AGP-01), melanoma (SK-MEL-19), lung carcinoma (A549), and a healthy human kidney strain (HEK-293).	[102]
	Hydroethanolic inflorescence extract	Cytotoxicity	MTT test and molecular docking against JAK1 and JAK2 proteins	Spilanthol	-Cytotoxicity against gastric cancer.	[103]
	Lyophilized ethanol extract from leaves and flowers	Healing	The calcaneal tendon of male Lewis rats was partially transected and treated at the site of injury with 64 mg of a topical application containing 20% *A. oleracea*	Not informed	-↑ in the molecular organization and content of collagen; -Potential application in tendon repair.	[67]
	Alkylamide-rich hexane fraction from flowers	Inflammatory pain	Swiss male adult mice pretreated, before the acute inflammatory response was induced by an injection of carrageenan into the right hind paw	Alkylamide	-↓ in the paw withdrawal threshold; -↓ in mechanical allodynia; -Effective and long-lasting antiallodynic and anti-oedematogenic activities; -↓ in MPO activity; TNF-α and IL-1β levels; SOD, CAT, and GSH contents; -Prevented the production of LOOH.	[104]
*Talinum triangulare*	Powder	Prebiotic	Chicks of the SASSO strain fed with feed +2% powder	Possible synergy between proteins and phytochemicals	-↑ in significantly lower mortality rates; -↔ between the red and white blood cell values; -↑ in the concentration of butyric, valeric, and heptanoic acids.	[105]
	Lyophilized aqueous extract	Antioxidant and anti-inflammatory	Matured male albino Wistar rats with AUC induced by 5% of DSS received 200 mg of lyophilized leaf/kg of body weight	Extract rich in phytochemicals	-↑ in body weight; -Ameliorated the toxic effect by length and weight of the colon; -↓ in the inflammatory marker’s levels in the colon; -It was more effective in inhibiting inflammation than sulphasalazine; -↓ in the colonic level of malondialdehyde; -↓ in H_2_O_2_ production; -↑ in the GSH and protein levels in the colon; -Reversed the DSS-induced inhibition of the cytoprotective enzymes.	[106]
*Pereskia bleo*	Methanolic, hexane, and chloroform extracts from leaves	Antioxidant and antibacterial activities	DPPH assay, MIC, and MBC	Not informed	-IC_50_ values between 33.83 and 379.41 µg/mL; -Strong inhibitory action for *Staphylococcus aureus*, *Streptococcus pyogenes*, *Pseudomonas aeruginosa*, and *Escherichia coli*.	[89]
*Bidens bipinnata*	Hydroalcoholic extract from aerial parts	Hepatoprotective effect	Administration of extract of B. bipinnata (50, 100, and 200 mg/kg) in mice with an acute liver injury for seven days	Not informed	-↓ in liver weight, serum transaminases, and hepatic morphologic changes; -↑ in SOD and glutathione peroxidase; -Suppressed nitric oxide production and nuclear factor-kappaB activation.	[107]
	Hexane extract from leaves, flowers, roots, stems, and fruit	Antifungal	Inoculation on YPD agar medium	Linoleic acid and dehydroabietic acid	-Inhibition of *C. albicans*, *C. glabrata*, *C. tropicalis*, *C. krusei*, and *C. orthopsilosis*.	[108]
*Costus spiralis*	Ethyl acetate fraction from methanolic extract of leaves	Antihyperglycemic properties	Enzyme assay	Schaftoside and isoschaftoside	-IC_50_ 1.95 times higher than acarbose.	[109]
	Aqueous extract from leaves and stems	Antioxidant activity and cytogenotoxic effects	DPPH assay and mitotic index; and the frequency of chromosomal aberrations, micronuclei, and nuclear abnormalities	Not informed	-IC_50_ 11.82 mg/mL to leaves and 15.38 mg/mL to stems; -Inhibitory effect on *Allium cepa* root’s growth.	[110]

Notes. ↔: no significant result; ↑: increase; ↓: decrease; DPPH: 2,2-diphenyl-1-picrylhydrazyl; ABTS: (2,2′-azino-bis(3-ethylbenzothiazoline-6-sulfonic acid); FRAP: ferric reducing/antioxidant potential; TE: trolox equivalent; FS, ferrous sulphate; ORAC: oxygen radical absorbance capacity; HOCl: hypochlorous acid scavenging activity; GI50: growth inhibition 50; II: intraperitoneal injection; DOCA: deoxycorticosterone acetate; NaCl: sodium chloride; MTT: (3-(4,5-dimethylthiazol-2-yl)-2,5-diphenyl-2H-tetrazolium bromide); JAK: Janus associated kinase; MPO: myeloperoxidase; TNF-α: tumor necrosis factor alpha; IL-1β: interleukin-1 beta; SOD: superoxide dismutase; CAT: catalase activities; GSH: glutathione; LOOH: lipid hydroperoxides; AUC: acute ulcerative colitis; DSS: dextran sodium sulphate; H_2_O_2_: hydrogen peroxide; IC_50_: medium inhibitory concentration; MIC: minimum inhibitory concentration; MBC: minimum bactericidal concentration; and YPD: yeast peptone dextrose.

In the literature, few studies have focused on identifying and quantifying phytochemical compounds, as well as investigating some biological properties of the extracts. More research is necessary, including studies on nutritional composition, metabolomics, and in vitro and in vivo assays.

### 2.6. Costus spiralis (Jacq.) Roscoe

The leaf of *Costus spiralis* (Figure 1F) is part of the Costaceae family and is a rhizomatous herb. In different regions of Brazil, it is also known as “pobre-velho”, “caninha-do-brejo”, and “cana-de-macaco”, among others, which vary according to the region of the country. Their morphology is characterized as herbs with a 11–15 cm long sheath, a bract, and a very characteristic reddish lip [111].

The population uses the stem bark part to treat digestive tract symptoms such as sore throat and fever [112]. However, its most promising use appears to be for urinary tract diseases [113]. Corroborating this, Carmona and Pereira [114] reported that the leaves of *C. spiralis* are used as a medicinal herb in Brazilian public health pharmacies. They are recommended for treating urinary diseases and for their diuretic effects. The leaves can be found in tincture, capsule, powder, and topical forms.

Few studies have been carried out on the chemical composition of *C. spiralis*, but they have shown that this species is a rich source of flavonoids. Hydroalcoholic extracts and their fractions (hexane, ethyl acetate, and methanol) identified glycosylated flavones of apigenin, including vicenin II and schaftoside [115]. Vicenin II has antioxidant, anti-inflammatory, anti-glycation, antinociceptive, antiproliferative, and hepatoprotective actions [116], while schaftoside exhibits anti-inflammatory activity and may inhibit the inflammatory cytokines IL-1β, IL-6, and TNF-α [117].

Some in vitro studies have indicated that *C. spiralis* is a promising herb for human health (Table 3). The hydroethanolic extract has been proven to benefit the urinary system in Wistar rats with cisplatin-induced nephrotoxicity, as demonstrated by Amorim et al. [115]. This extract, containing C-glycosylated apigenin flavone, was safe and responsible for significantly decreasing plasma creatinine concentration. In addition, it increased urinary excretion and water intake, which, according to the authors, was possibly due to the proposed renal protection mechanism for the extract.

Another study tested different extracts of this leaf in adult Swiss mice and observed a decrease in paw licking time and paw edema. These results demonstrate the potential of *C. spiralis* as a promising anti-inflammatory and peripheral nociceptive [118]. Recently, Duarte et al. [78] conducted a study on the effects of administering *C. spiralis* leaf powder and methanolic extract (rich in the flavonoid guaijaverin) in male Wistar rats. The findings indicated that the powder had a significant time-dependent hypoglycemic effect. Both tested samples decreased the plasma levels of LDL-c, non-HDL cholesterol, and malondialdehyde without affecting the kidney and liver functions. Therefore, this study indicates that *C. spiralis* may have a beneficial effect on glycemic and lipid metabolism.

## 3. New Technologies

### 3.1. FoodTech

The innovative Food Technology (FoodTech or FT) scenario has emerged as a powerful ally in harnessing the unexplored potential of the Amazonian UFPs [119]. FT is an ecosystem comprising agri-food companies and startups that apply technologies, innovation, and science in the food sector to create efficiency, sustainability, and healthiness in the design, production, selection, delivery, and use of food, packaging, supplements, additives, and others [106]. The convergence of technology, innovation, and science for the UFP sector offers a transformative path to increase the efficiency, sustainability, and health benefits associated with these unique botanical resources [119]. FT covers areas such as biotech agriculture, trading platforms, bioenergy and biomaterials, robotics, green food, and new farming systems, as well as disruptive technologies such as the Internet of things (IoT), big data, Artificial Intelligence (AI), nanotechnology, “omics” technologies, bioinformatics, genome sequencing and systems biology, and other automation solutions [120,121]. These technologies can contribute to resolving the complexities and challenges inherent to research into UFPs and markets in the Amazon [119]. Some of the main types of FT are shown in the Supplementary Material, Figure S1.

The relevance of FT for the food sector includes (i) greater productivity and less waste of natural resources and energy, (ii) better traceability and monitoring of processes and ingredients used, in addition to identifying recalls or disease outbreaks along the production chain, (iii) sustainable agricultural practices, (iv) the creation of personalized diets adapted to individual preferences and needs, (v) the adoption of blockchain for efficient supply chain management, and (vi) the creation of healthier foods and sustainable alternatives that promote health and well-being [122]. Examples of FT already established in the market include startups specializing in alternative proteins, nutraceuticals, agricultural robotics, FoodService in the Metaverse, 3D food printers, cellular agriculture, etc. [123]. In the context of Amazonian UFPs, the applications of these technologies could transform the research and commercialization of these plants. For example, food processing technologies such as high-pressure sterilization (HPP) [124] and pulsed light pasteurization (PLP) [125] could be used to preserve the bioactive properties of plants without compromising their nutritional integrity, facilitating the development of new functional products from these species [125,126]. Furthermore, the use of AI and data analytics can optimize the cultivation and harvesting of Amazonian UFPs, monitoring environmental and predictive conditions to ensure more consistent and sustainable production. Sustainable agricultural practices promoted by FT, such as precision agriculture and agroecology, could be adapted for the Amazon, ensuring more efficient and ecological exploitation of UFPs, while urban and vertical farming techniques could make it possible to grow these plants in urban areas, promoting food diversification in regions where these species are not traditionally cultivated [127]. The integration of blockchain technology could also enhance supply chain transparency and traceability [128], promoting consumer trust and facilitating the ethical sourcing of Amazonian UFPs.

Although investments exceed USD 1 billion in Brazilian FoodTech and Brazil is a megabiodiverse country (i.e., with more than 50,000 native plant species) [7,122], no FT has yet been agreed specifically for Amazon UFPs, and this gap represents an unexplored opportunity for innovative solutions that add value and boost the region’s bioeconomy. It is important to distinguish between food companies, which have incorporated some technology into their processes, and FT, which has a greater focus on technology and innovation, with disruptive solutions to the challenges of the food industry. In this context, FT could leverage the chain of Amazonian UFPs, developing products with high added value (e.g., bioactive peptides, fixed or essential oils, antioxidants, pigments, vitamins, functional sugars, resistant starch, probiotics, etc.), using innovative techniques, such as green extraction methods (e.g., high pressure, supercritical fluid, electroscopy, electrical pulses, etc.), recent drying technologies (e.g., microwave, freeze-drying, infrared, vacuum impregnation, etc.), and controlled delivery methods (e.g., nano- or microencapsulation, etc.) [129,130]. In addition, FT could provide resources and technological solutions to overcome the challenges of scalability, seasonality, low availability, organoleptic characteristics, and the costly processes in developing products with UFPs [131].

FT that specializes in plant-based technology can find in UFPs an opportunity to explore new ingredients and foods, to promote a more diversified, sustainable, and healthy diet [5]. Incorporating UFPs in plant-based diets increases the intake of different limiting amino acids and improves the quality of ingested proteins [70]. Another study showed that *Moringa oleifera*, *Jatropha curcas*, *Pereskia aculeata*, *Beta vulgaris*, and *Bambusa vulgaris* have great industrial potential due to their potential as a source of proteins, mainly in the leaves, stems, and seeds, with contents ranging from 20 to 37% [5]. *P. bleo* or *Leuenbergeria bleo* has also been reported to have 30% of protein, an option for vegetarian and vegan diets [8].

More broadly, FT can offer technological solutions to implement sustainable agricultural practices of UFPs in the Amazon region through AgriTech (i.e., agricultural technology companies), such as agroecology, permaculture, and organic agriculture, in addition to encouraging crop rotation, intercropping, and integrated pest management, using resources such as remote sensors, drones and satellite images, and cloud and quantum computing to assist in the efficient and sustainable management of cultivation areas [122]. The development of FT using Amazonian UFPs should also create opportunities for local producers (e.g., technical training, access to adequate resources and technologies, incentives for sustainable production, family farming, and trade), contributing to the appreciation and preservation of traditional knowledge and the socioeconomic development of the communities involved [132].

Therefore, initiatives combining Agri-FoodTech and Amazonian UFPs can add economic value, expand the offer of healthy and sustainable options, and boost the economy and the market [122].

### 3.2. Market

There is a growing global demand for sustainable food with unique characteristics, which can open up promising possibilities for the national and international markets of Amazonian UFPs. Despite the appreciation in recent years for organic and traditional products from Brazil’s biodiversity, the market for Amazonian UFPs is still relatively scarce and complex, influenced by market relations, the political environment, the resource involved, and other actors. Most companies focus on producing powders using traditional drying methods. This may result from limited knowledge about the nutritional, technological, and sensory properties of UFPs, in addition to insufficient public policies. In addition, there are challenges in the supply chain due to seasonality and the lack of standardization of production and supply [7].

On the other hand, regulatory issues can also be a bottleneck due to the lack of comprehensive in vivo and clinical studies on nutritional and toxicity properties, which can generate uncertainties and require more outstanding evaluation by the regulatory sectors [133,134]. However, there are currently initiatives by researchers, entrepreneurs, and institutions related to the advancement of the Amazonian UFP market, such as Embrapa [135], *Rede Inovativa* [136], Seed—Startups and Entrepreneurship Ecosystem Development [137], and Imazon [138]. Therefore, only with continuous efforts will it be possible to value the Amazonian UFP market by promoting market chairs that promote diversity and sustainability.

### 3.3. Challenges and Future Trends for Food Technology Using UFPs

Research in Brazil should provide the theoretical base and develop mechanisms to increase the visibility of Amazonian UFPs, especially those that are known, consumed, and marketed in an incipient way, since the popularization of these vegetables can strongly contribute to income generation and jobs [54]. This includes investments to improve the scientific knowledge of nutritional composition, sensory characteristics, processing methods, product development, and clear and adequate regulations.

It is essential to establish commercial chains for Amazonian UFPs, and this includes industry 4.0 topics, such as technologies involving genomic engineering, RNA interference (RNAi), blockchain tracking, the Internet of things (IoT), and geographic information systems to monitor and ensure the origin, quality, and sustainability, as well as AI and machine learning to optimize the species selection and the prediction of nutritional properties, formulations, diet customizations, and more. In addition to biotechnological and digital technologies, advanced processing methods (e.g., green extraction, microencapsulation, etc.) can also be employed to preserve nutrients, improve stability, and diversify, in addition to adding market value to UFPs [54,122,130].

On the other hand, accessible technologies such as social media, blogs, mobile apps, and educational videos can share recipes, preparation tips, and nutritional information to promote the broader adoption of the cultural and gastronomic value of Amazonian UFPs, which can generate greater experimentation and combat resistance to changing eating habits [54]. In addition to food sectors, UFPs can also be used in innovations in medical–pharmaceutical research for pharmaceuticals, medicines, and cosmetics and innovations related to materials science for solutions and the application of materials of plant origin, among others. Therefore, through integrated and collaborative approaches, Amazonian UFPs can play an important role in promoting sustainability, income generation, and dietary diversification, contributing to the preservation of biodiversity and the well-being of the communities involved.

## 4. Conclusions

The incalculable wealth of natural and genetic resources in the Amazon region can provide support for the development of value-added biotechnological processes and products that include the inclusive social process of sustainable exploitation, boosting the bioeconomy. From this perspective, Amazonian UFPs are a true “culinary treasure trove”. These foods represent a more sustainable and nutritionally sound concept that can increase food sovereignty and nutritional security from both a collective and consumer point of view. In addition to being sources of essential nutrients, they provide various bioactive components that can contribute to metabolic processes, offer health benefits, and open up a range of possibilities in the fields of food science, nutrition, and health and in the economic development of the region.

This review highlights the lack of comprehensive nutritional composition data for UFPs. For instance, there is a shortage of information regarding the macronutrient and micronutrient content of *B. bipinnata*, a native Brazilian species encouraged for acquisition and purchase under public policies promoting family farming, such as the National School Feeding Program (PNAE) and the Food Acquisition Program (PAA). Similarly, there is limited knowledge about the proximal, mineral, and vitamin compositions of *P. bleo* and *C. spiralis*. Only *X. sagittifolium*, *A. oleracea*, and *T. triangulare* have available data on dietary fiber. In addition, the absence of investigations into the presence of carotenoids (despite their potentially valuable antioxidant properties) in *P. bleo*, *C. spiralis*, and *B. bipinnata* is notable. Although all the species have been examined for their biological effects, the scarcity of in vivo trials signifies a lack of comprehensive study into the functional properties of these plants.

There is limited knowledge about the potential of these species, but some findings are noteworthy. For instance, spilanthol, the main bioactive compound extracted from *A. oleracea*, has drawn the attention of researchers in the food and pharmaceutical industries because of its biological properties. The increasing interest in this compound has led to the commercial availability of spilanthol extract, which can currently be purchased on websites.

This review emphasizes the importance of conducting more research into chemical composition, bioaccessibility, and bioavailability, as well as carrying out in vivo trials, including toxicity evaluations of the extracts. These studies are essential for assessing their potential contribution to human health. Additionally, using green extraction processes to preserve nutrients and researching microencapsulation can provide valuable insights for developing products and processes for the consumer market. This will not only add value to these species but also encourage their consumption and cultivation. The appreciation and preservation of Amazonian UFPs heavily rely on scientific and technological advancements. Collaborative efforts among researchers, companies, and public policies can help to increase the cultivation and consumption of UFPs, promoting healthier and more sustainable eating habits.

## Data Availability

No new data were created or analyzed in this study. Data sharing is not applicable to this article.

## Supplementary Materials

The following supporting information can be downloaded at: https://www.mdpi.com/article/10.3390/foods13182925/s1, Figure S1: Main types of Food Tech.
