# Supplementary material for: Unconventional Edible Plants of the Amazon: Bioactive Compounds, Health Benefits, Challenges, and Future Trends
Source: Foods. 2024 Sep 15;13(18):2925. doi: 10.3390/foods13182925 (PMC11431067; doi:10.3390/foods13182925)
Supplement: Supplementary file 1 [file foods-13-02925-s001.zip › foods-3189066-supplementary.pdf]

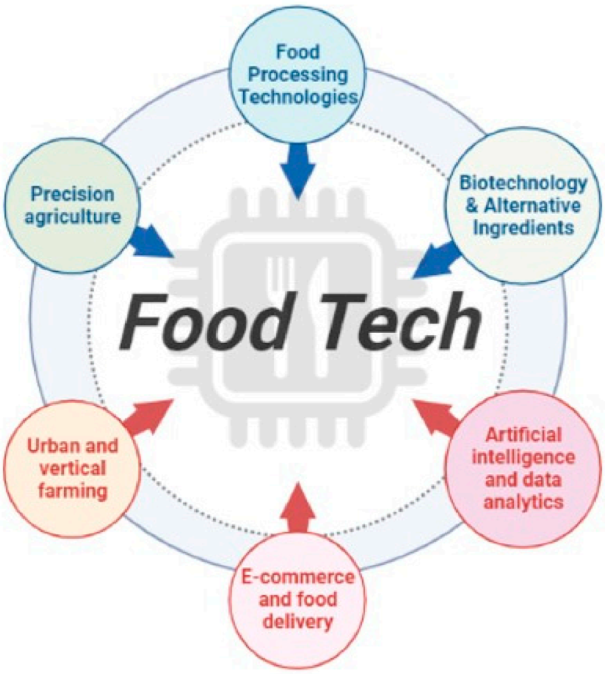

Supplementary Material Figure S1. Main types of Food Tech: (i) Precision agriculture: uses technologies such as sensors, drones, and data analysis to optimize agricultural production, monitor soil and plant conditions, improve efficiency in the use of resources, and make decisions based on data to increase productivity. (ii) Food processing technologies: encompasses a variety of technologies to improve food processing, preservation, and packaging, such as advanced thermal processing techniques such as high-pressure sterilization (HPP) and pulsed light pasteurization (PLP), in addition to innovative packaging methods such as active and smart packaging. (iii) Biotechnology and alternative ingredients: involves the production of alternative proteins, such as laboratory-grown meat, plant-based dairy products, and egg substitutes, as well as using microorganisms to produce functional ingredients and food additives. (iv) Artificial intelligence and data analysis: uses artificial intelligence algorithms and data analysis to improve operational efficiency, optimize the supply chain, customize food products, predict market trends, and improve the consumer experience. (v) E-commerce and food delivery: these are ordering applications and logistics solutions to improve the convenience and accessibility of food. (vi) Urban and vertical agriculture: bringing food production to urban areas, using techniques such as hydroponics, aeroponics, and vertical agriculture indoors, allowing local fresh food production in limited spaces.
